# Revisiting dispersible milk-drug tablets as a solid lipid formulation in the context of digestion

**DOI:** 10.1016/j.ijpharm.2018.10.069

**Published:** 2019-01-10

**Authors:** Syaza Y. Binte Abu Bakar, Malinda Salim, Andrew J. Clulow, Adrian Hawley, Ben J. Boyd

**Affiliations:** a Drug Delivery, Disposition and Dynamics, Monash Institute of Pharmaceutical Sciences, Monash University, 381 Royal Parade, Parkville, Victoria 3052, Australia; b SAXS/WAXS Beamline, Australian Synchrotron, ANSTO, 800 Blackburn Road, Clayton, Victoria 3169, Australia; c ARC Centre of Excellence in Convergent Bio-Nano Science and Technology, Monash Institute of Pharmaceutical Sciences, Monash University, 381 Royal Parade, Parkville, Victoria 3052, Australia

**Keywords:** API, active pharmaceutical ingredient, GIT, gastrointestinal tract, TAG, triacylglycerol, DAG, diacylglycerol, MAG, monoacylglycerol, FFA, free fatty acid, DC, direct compression, SAXS, small-angle X-ray scattering, Dispersible tablet, Milk, *In vitro* digestion, X-ray scattering, Drug solubilisation, Self-assembly

## Abstract

Oral delivery of dispersible tablets is a preferred route of administration for paediatrics due to ease of administration and dose control. Milk has gained interest as a drug delivery system due to its ability to dissolve poorly water-soluble drugs. There are no reports of milk tablet formulations being assessed in the context of lipid digestion, which is critical in influencing orally administered drug solubility and bioavailability. Milk-drug tablets were formulated by blending freeze-dried bovine milk or infant formula with the poorly water-soluble drug cinnarizine, which were directly compressed. Tablet strength, friability and dispersibility were quantified and synchrotron X-ray scattering was used to determine the lipid liquid crystalline phases formed during *in vitro* digestion of dispersed tablets and their effects on drug solubilisation. Tableting had a significant impact on the self-assembly of lipids in redispersed milk tablets whereas no effect was seen for infant formula tablets. Incorporation of the disintegrant poly(vinylpolypyrrolidone) to reduce tablet dispersion times promoted the formation of hexagonal liquid crystalline phases upon digestion but had minimal effect on drug solubilisation. These findings show that similar to the use of liquid milk, the formulation of milk-drug tablets can be used to improve solubilisation of poorly water-soluble drugs.

## Graphical abstract

**Figure:**
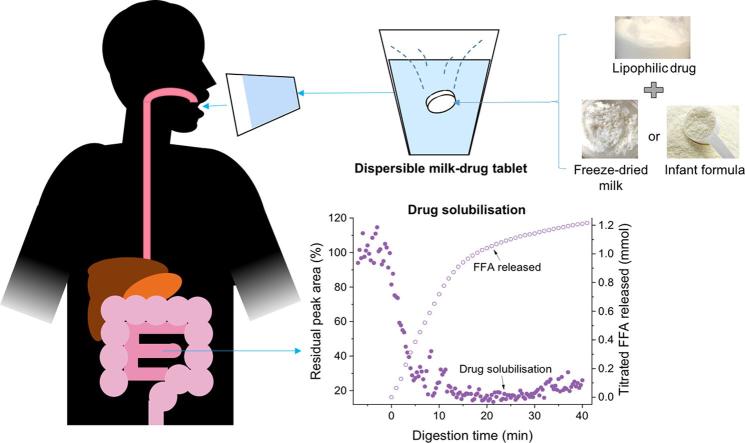

## 1 Introduction

Paediatric patients are often administered medications that are suited for the adult population. This includes swallowing a tablet as a whole, which might pose as an obstacle for toddlers and infants or liquid oral formulations that could lead to variability in doses given by the caretaker (Nunn and Williams, 2005, Davies and Tuleu, 2008). Manipulation of the medication such as crushing the tablet or extemporaneous compounding can potentially increase the risk of overdosing and underdosing, thus reducing efficacy (WHO, 2010). Safe and effective alternatives are therefore required to ensure that medications are appropriate following the age, weight and physiological condition of the child.

Oral delivery of dispersible tablets is regarded as a preferred route of administration for paediatrics. The variability involved in breaking the tablet into smaller pieces, increasing the likelihood of affecting the dose, would be averted as the desired dose can be controlled by the reconstituted volume for administration. Although the excipients in dispersible tablet formulations are regarded as safe for consumption by adults, this does not necessarily translate to paediatrics (Krause and Breitkreutz, 2008).

Milk is a natural commodity and serves as an optimal form of nourishment for infants up to the age of six months for growth and development. Milk contains 3 to 6 *w/v*% fat, of which the triacylglycerols (TAGs) comprise around 98 *wt*% of the total milk fat content (Jensen, 1995, Liu et al., 2017). This interesting bioemulsion has gained interest in recent years as a lipid-based formulation to enhance the solubilisation of poorly water-soluble drugs (Kamal et al., 2016). While previous studies have suggested that dispersing a drug in native milk can increase the drug solubility (Macheras and Reppas, 1986, Macheras et al., 1990, Topaloğlu et al., 1999), little research has been directed at understanding the effect of digestion on drug solubilisation, particularly for dispersible solid dosage forms. In the case of poorly soluble lipophilic drugs, digestion is a crucial component as the nature of the lipids and their digestion products promotes drug solubilisation and transport to the systemic circulation (Pouton, 2006, Feeney et al., 2016, Kaukonen et al., 2004, Mu et al., 2013). It has been recently shown that the digestion of milk can be a particularly effective way to drive solubilisation of co-administered drug (Salim et al., 2018).

While milk is regarded as safe for consumption by adults and paediatrics, this commodity has not been approved formally as a pharmaceutical excipient by the Food and Drug Administration for a number of reasons, with primary reasons being chemical consistency and accessibility. Although infants receive mother’s milk or infant formula from their carers and adults could simply drink a glass of milk, the types of milk available and accessibility to different kinds of milk vary depending on the socioeconomic, societal and geographical circumstances. In addition, the fatty acid composition, which is critical to its interaction with drugs upon digestion, is highly variable between types of milk and even day-to-day from the same species presenting a major regulatory hurdle to its use as a formal excipient in drug formulations (Liu et al., 2017).

**Fig. 1:**
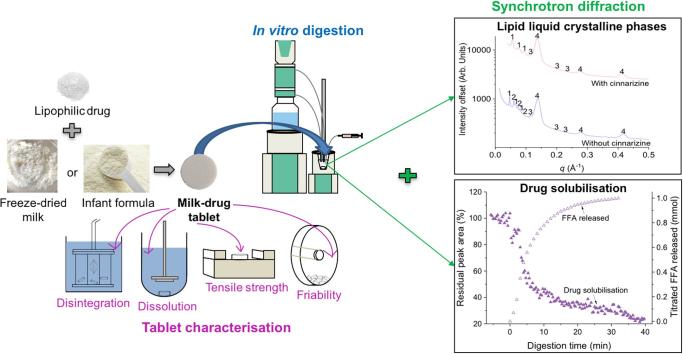
Formulation of dispersible freeze-dried milk/infant formula-drug tablets using freeze-dried milk or infant formula and cinnarizine as the drug. Tablet characteristics were studied using pharmacopeial methods, while lipid self-assembly and drug solubilisation during digestion were studied using *in situ* X-ray diffraction.

To address these problems, blending the drug with locally available milk would minimise issues relating to accessibility and furthermore freeze-drying the milk could potentially eliminate the issue of maintaining cold-chain storage for liquid milk. Infant formula was also used in this study to remove the issue of temporal variability in milk fat composition. The use of direct compression (DC) to generate these tablets is less complex than other tableting techniques enabling easier manufacturing and accessibility in developing countries. Therefore, this study aims to add to the understanding of the role of milk and infant formula as a lipid-based formulation by investigating its behaviour in formulation as dispersible freeze-dried milk/infant formula-drug tablets, illustrated schematically in Fig. 1. Cinnarizine, a poorly water-soluble antihistaminic drug was chosen as a model drug due to its moderately lipophilic character. The freeze-dried milk/infant formula-drug solid dosage forms were characterised by uniformity of weight, friability, tensile strength, disintegration and dissolution time. Real-time monitoring of lipid liquid crystalline phases formed during *in vitro* digestion of the dispersible tablets was conducted using synchrotron small-angle X-ray scattering (SAXS) both in the presence and absence of cinnarizine and added disintegrant. Finally, SAXS was used to determine the scattering intensity of diffraction peaks from crystalline cinnarizine to track its solubilisation during digestion.

## 2 Materials and methods

### 2.1 Materials

Full fat bovine milk (3.8 *w/v*% fat) was purchased from Coles supermarkets (Brunswick or Mt Waverly, Victoria, Australia) and powdered infant formula was kindly donated by Medicines for Malaria Venture (MMV, Switzerland). The nutritional information for milk and the infant formula are provided in Table 1. Poly(vinylpolypyrrolidone) (PVPP), trizma®-maleate (reagent grade) and cinnarizine powder (≥99% purity) were purchased from Sigma Aldrich (St. Louis, MO, USA). Calcium chloride dihydrate (>99% purity) and sodium hydroxide pellets (>97% purity) were obtained from Ajax Finechem (Seven Hills, NSW, Australia). Hydrochloric acid (36%) was purchased from LabServ (Ireland). Sodium chloride (>99.7% purity) was purchased from Chem Supply (Gillman, SA, Australia). Sodium azide (>99% purity) was obtained from Merck (Darmstadt, Germany). USP grade pancreatin extract was obtained from Southern Biologicals (Nunawading, Victoria, Australia). Unless otherwise stated, all chemicals were used without further purification and water was acquired from Merck Q-POD Ultrapure Water Remote Dispenser (Darmstadt, Germany).

**Table 1:** Nutritional information of full fat bovine milk and infant formula.

	Full fat bovine milk		Infant formula
Average quantity	per 100 mL	per 100 g	per 100 g
Protein	10.2 g	9.9 g	10.8 g
Fat – Total	3.8 g	3.7 g	27.1 g
– Saturated	2.5 g	2.4 g	15 g
Carbohydrate	4.8 g	4.7 g	56 g
– Sugars	4.8 g	4.7 g	8.3 g
Vitamin A	41 µg	40 µg	425 µg
Vitamin E	–	–	8.5 mg

### 2.2 Tablet formulations

*Freeze-dried milk* Bovine milk (5 mL) was added into a 20 mL glass scintillation vial and freeze-dried for two nights. The freeze-dried milk (585 mg) was weighed into a 20 mL glass scintillation vial using an analytical balance. Typically, 30 × 5 mL portions of milk were freeze-dried at a time to produce 30 tablets from 150 mL of milk.

*Freeze-dried milk-cinnarizine* Bovine milk (5 mL) was added into a 20 mL glass scintillation vial and freeze-dried for two nights. Freeze-dried milk (585 mg) and cinnarizine powder (13 mg) were weighed separately using an analytical balance before being added into a 20 mL glass scintillation vial. The powders were then mixed thoroughly for 5 min with a vortex mixer. Typically, 30 × 5 mL portions of milk were freeze-dried at a time to produce 30 tablets from 150 mL of milk.

*Freeze-dried milk-cinnarizine-PVPP* Bovine milk (5 mL) was added into a 20 mL glass scintillation vial and freeze-dried for two nights. Freeze-dried milk (585 mg), cinnarizine powder (13 mg) and PVPP (179 mg) were weighed separately using an analytical balance before being added into a single glass vial. The powders were then mixed thoroughly for 5 min with a vortex mixer. Typically, 30 × 5 mL portions of milk were freeze-dried at a time to produce 30 tablets from 150 mL of milk.

*Infant formula* Using an analytical balance, the infant formula powder (701 mg) was weighed into a 20 mL glass scintillation vial.

*Infant formula-cinnarizine* Using an analytical balance, infant formula powder (701 mg) and cinnarizine powder (13 mg) were weighed into a 20 mL glass scintillation vial and thoroughly mixed for 5 min using a vortex mixer.

*Infant formula-cinnarizine-PVPP* Using an analytical balance, infant formula powder (701 mg) , cinnarizine powder (13 mg)and PVPP (214 mg) were weighed into a 20 mL glass scintillation vial and thoroughly mixed for 5 min using a vortex mixer.

*Tablet compression* The blended formulations were tableted directly after vortex mixing. Magnesium stearate (0.5 *w/w*% to tablet) was added to lubricate the tablet press (Gamlen Tableting Ltd, Nottingham, United Kingdom) before compressing the tablets with a compression force of 60 kg at 5 mm/min fitted with a 14 mm diameter flat-faced bevelled edge.

### 2.3 Tablet analysis

#### 2.3.1 Uniformity of weight and tablet thickness

According to USP <905>, the uniformity of tablet weight was measured for 10 tablets, which were chosen randomly and weighed individually on an analytical balance. Acceptable tablet weight relative to the target masses in Table 2 was mass ±5%. The thickness of each tablet (n = 10) was obtained using a pair of Vernier calipers.

**Table 2:** Composition of freeze-dried milk/infant formula-drug tablets.

Lipid source	Milk/Formula (mg)	Cinnarizine (mg)	30 *w/w*% PVPP (mg)	Target mass without PVPP (mg)	Target mass with PVPP (mg)	Amount of drug (mg)/g of fat
Bovine milk	585	13	179	597	777	66
Infant formula	701	13	214	713	927	66

#### 2.3.2 Breaking force test

Breaking force of the tablets was performed (n = 10) using a hardness tester (Electrolab, Mumbai, India) as described in USP <1217>. The tensile strength of the tablets was then determined using the Fell and Newton equation (Eq. (1)):

(1) $$\sigma_{t}\left(MPa\right)=2F/\pi DT$$

where $\sigma_{t}$ is tensile strength, F is crushing force (N), D is tablet diameter (mm) and T is tablet thickness (mm).

#### 2.3.3 Friability test

Similarly, the friability test (USP <1216>) was carried out on tablets using a friability testing apparatus (Erweka TAR-Series Frankfurt, Germany). Approximately 6.5 g of tablets (if average mass of individual tablet was <650 mg) or approximately 4 g of tablets (if average mass of individual tablet was >650 mg) (initial weight, *W_i_*) were placed into the friability apparatus and rotated at 25 rpm for 4 min. The tablets were then de-dusted and re-weighed (final weight, *W_f_*) and if any of the tablets were cracked, cleaved or broken, the test was considered as failed. The friability was calculated from Eq. (2):

(2) $$\mathrm{Friability}\left(\%\right)=[\left(W_{i}-W_{f}\right)/W_{i}]\times 100\%$$

#### 2.3.4 Disintegration test

Disintegration tests were conducted using a disintegration apparatus (Electrolab, Mumbai, India) in accordance with USP <701>. One tablet was placed in each of the six tubes of the basket and the assembly was suspended in a 1 L beaker containing distilled water. The temperature was maintained at 37 ± 2 °C. The test was considered successful once the tablets had disintegrated completely.

#### 2.3.5 Dissolution test

The release rate of cinnarizine from the compressed tablets (n = 3 for each type of tablets) was determined using the paddle apparatus USP type II (Klausen Trading Company Pty Ltd, Victoria, Australia). The dissolution test was conducted in 900 mL of Milli-Q water as the dissolution medium at 37 ± 0.5 °C at 75 rpm in accordance with USP  <711>. A 3 mL aliquot was withdrawn at time intervals of 1, 2, 5, 10, 20 and 30 min and no large solid particles were observed to be drawn into the 3 mL syringe. The withdrawn aliquots were then replaced with 3 mL of media. No filtration or sample pre-treatment was performed prior to measuring the absorbance of the aliquots. The absorbance was measured at a wavelength of 600 nm using the EnSpire® Multimode UV plate reader (PerkinElmer, USA).

### 2.4 Particle size measurements

Particle size distributions of the fat globules from the freeze-dried milk/infant formula solid dosage forms were measured after reconstitution/dispersal in water by laser light scattering using a Mastersizer-S (Malvern Panalytical, United Kingdom), equipped with a He-Ne laser of wavelength 633 nm and a 300-RF lens for the detection of size ranging from 0.05 to 880 μm. The particle density of the milk fat globules was taken to be 0.92 g/cm^3^ and the refractive indexes of milk and water were taken to be 1.462 and 1.330, respectively (Michalski et al., 2001). Water (50 mL) was added to a Malvern Panalytical dispersion unit and the redispersed samples were added to reach an obscuration value between 10 and 15% prior to the measurements. The volume-weighted mean diameter of the particles was then recorded as D_4,3_ generated by the in-built instrument software based on calculated size distributions by volume.

### 2.5 *In vitro* digestion

*In vitro* lipolysis and SAXS measurements were performed on the freeze-dried milk/infant formula-drug tablets using methods described previously (Williams et al., 2012, Salentinig et al., 2013). For each digestion experiment, 4 tablets were reconstituted in 50 mM trizma-maleate digestion buffer (final volume 20 mL) at pH 6.5, which also contained 5 mM calcium chloride, 150 mM sodium chloride and 6 mM sodium azide. Calcium chloride was added to remove any free fatty acids that could potentially surround the milk fat globule thereby enabling the digestions to progress. The samples were vortexed until thoroughly mixed prior to being transferred into a thermostatted glass vessel (maintained at a constant temperature of 37 °C) connected to a pH stat auto titrator (Metrohm® AG) under constant magnetic stirring. The apparatus was connected to a computer and operated using Tiamo 2.0 software (Metrohm®). Tablets comprising freeze-dried milk or infant formula were redispersed in 10 mL of digestion buffer. The dispersed milk or infant formula formulation were made up with additional digestion buffer in a 20 mL volumetric flask before being loaded into the digestion vessel. Prior to the injection of lipase, the pH of the sample was adjusted using HCl or NaOH to a value of 6.500 ± 0.003. Freeze-dried pancreatic lipase was prepared in the digestion buffer and 2.25 mL was injected into the sample vessel to give an activity of around 700 TBU/mL of digest (measured independently by adding 2 mL of reconstituted lipase solution to 6 g of tributyrin stirred vigorously with 18 mL of digestion buffer).

Throughout the digestion of the milk and infant formula lipids, either 0.2 M or 2.0 M NaOH was added to maintain the pH at 6.5 to counter the decrease in pH as a result of the liberation of fatty acids. 0.2 M NaOH was used to determine the kinetics of digestion as a function of time while 2.0 M NaOH was used for SAXS measurements of drug solubility to minimise dilution of the digesting samples. Based on the volume of NaOH required to maintain the pH at 6.5, the amount of titrated (ionised) fatty acids was determined following the subtraction of volume of NaOH from a blank digestion (digestion buffer only with no added lipids). After an hour of digestion, the pH of the digested milk lipids was increased to pH 9.0 using NaOH (‘back titration’), for which the molar amount of NaOH required corresponds to the amount of unionised fatty acids at the end of digestion. Together with the amount of ionised fatty acids determined earlier, the total amount of fatty acids released during digestion was calculated. Subsequently, the extent of digestion was calculated using Eq. (3) and the estimated theoretical amount of fatty acids in milk was estimated to be around 2.16 mmol from prior literature reports (Soyeurt et al., 2006) assuming that 1 mol of TAG generates 2 mol of FFA using Eq. (3):

(3) $$\mathrm{Extent}\;of\;digestion\;\left(\%\right)=\frac{\mathrm{Ionised}\;FFA\;\left(mol\right)+Unionised\;FFA\;\left(mol\right)}{\mathrm{Theoretical}\;FA\;in\;milk\;\left(mol\right)}\times 100\%$$

### 2.6 Small angle X-ray scattering (SAXS): flow through measurements

SAXS experiments were carried out on the SAXS/WAXS beamline at the Australian Synchrotron (ANSTO, Clayton, Victoria) (Kirby et al., 2013) to determine real-time lipid liquid crystalline formation in milk/infant formula and the scattering intensity of cinnarizine. The *in vitro* digestion apparatus described above was coupled to the SAXS/WAXS beamline as described previously (Warren et al., 2011).

A peristaltic pump was used to move the contents of the glass digestion vessel at a flow rate of ~10 mL/min via silicon tubing through a 1.5 mm diameter quartz capillary mounted in the X-ray beam. Pancreatic lipase was added remotely using a syringe driver. An X-ray beam with a photon energy of 13 keV (wavelength, λ = 0.954 Å) was utilised in this investigation. Two different sample-to-detector distances of around 1.6 m (approximate *q* range of 0.01 < *q* < 0.67 Å^−1^) and 0.6 m (approximate *q* range of 0.04 < *q* < 2.00 Å^−1^) were used to monitor the liquid crystalline structures in milk/infant formula and the intensity of the drug diffraction peaks, respectively. *q* refers to the length of the scattering vector or momentum transfer and expressed as in Eq. (4):

(4) q=(4π/λ)sin(2θ/2)

where 2θ is the scattering angle. 2D SAXS patterns were recorded using a Pilatus 1 M detector (active area 169 × 179 mm^2^ with a pixel size of 172 µm), with a 5 s acquisition time and 15 s delay between each measurement. The phases of the liquid crystalline structures were identified from their characteristic diffraction peaks and the presence of crystalline drug was monitored using the intensity of characteristic diffraction peaks as a function of time. 2D diffraction patterns recorded were converted into a 1D plot of intensity of scattered X-rays against scattering vector (*q*) using the in-house developed software *Scatterbrain*. In the 1D profiles, the presence of lamellar, hexagonal and cubic liquid crystalline phases were determined based on the spacings of the diffraction peaks (Hyde, 2001).

Subsequently, the lattice parameter or physical dimension of unit cells in the crystal lattice, a, were calculated based on the interplanar spacing between the lattice planes (d_*hkl*_ = 2π/*q_hkl_*) using Eq. (5), where *hkl* are the Miller indices of each lattice plane.

$${Cubic:d}_{hkl}=\frac{a}{\sqrt{h^{2}+k^{2}+l^{2}}}$$

(5) $${Hexagonal:d}_{hkl}=\frac{a}{\sqrt{\frac{4}{3}\left(h^{2}+k^{2}+hk\right)+l^{2}{\left(\frac{a}{c}\right)}^{2}}}$$

## 3 Results

### 3.1 Characteristics of tablets

Our group has previously shown the role of milk digestion on the solubilisation of a drug and its polymorphism (Salim et al., 2018). In this study, we aim to understand the effect of digestion of milk and infant formula lipids on the solubilisation of a drug when presented as a solid dosage form. Prior to determining the effect of digestion on drug solubilisation, the properties of these freeze-dried milk/infant formula-drug tablets were evaluated.

#### 3.1.1 Uniformity of weight and thickness

**Fig. 2:**
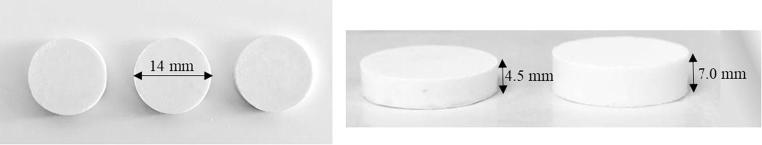
(a) Appearance of freeze-dried milk/infant formula-cinnarizine tablets and (b) thickness of freeze-dried milk tablets, left = without PVPP, right = with PVPP.

Table 3 summarises the blend characteristics and results of the tablet formulations. All tablets displayed minimal variation in weight and presence of disintegrant where the standard deviation of each batch was found to lie within ±5% of the target weight. The thickness of the tablets was found to be 4.5 to 9.0 mm (Fig. 2b).

**Table 3:** Properties of freeze-dried milk/infant formula-drug tablets with and without the disintegrant, PVPP (Data are mean ± SD, n = 10.

Lipid source	Amount of drug (mg)/g of fat	Diameter (mm)	Weight (mg)	Thickness (mm)	Tensile strength (MPa)	Friability (%)	Disintegration time (min)
*Tablets without PVPP*							
Bovine milk	66	14.00 ± 0.02	596 ± 4	4.51 ± 0.05	1.14 ± 0.02	0.6 ± 0.3	41.4 ± 1.3
Infant formula	66	14.00 ± 0.01	719 ± 3	5.50 ± 0.06	0.41 ± 0.01	2.2 ± 0.1	33.3 ± 1.1

*Tablets with 30 w/w% PVPP*							
Bovine milk	66	14.00 ± 0.02	777 ± 4	7.02 ± 0.07	1.04 ± 0.04	0.3 ± 0.3	21.5 ± 0.9
Infant formula	66	14.00 ± 0.01	927 ± 3	9.01 ± 0.06	0.43 ± 0.05	1.9 ± 0.2	6.2 ± 0.7

#### 3.1.2 Breaking force test

Tablets are required to withstand the severities of handling and transportation from the manufacturing sector until the tablet lands in the hands of consumers. To determine the resistance of tablets to mechanical strength applied, breaking force and friability tests were conducted. In this study, it was found that the tensile strength and friability of the tablets were largely influenced by the type of lipids present. Typically, tablets with tensile strengths of ≥0.8 to 1 MPa are considered as acceptable due to a lower probability of breaking the tablets (Kushner et al., 2014). Here, the tablets fall slightly outside the given range with average tensile strengths ranging from 0.41 to 1.14 MPa, with the freeze-dried milk-cinnarizine tablets displaying greater tensile strengths than infant formula-cinnarizine tablets. It has been reported that the physicochemical properties of the tablets are highly dependent on the binding agent used in the formulation. For instance, milk in dairy products were able to increase cohesion in complex matrices and fat components promote the formation of bonds between particles (Özkan et al., 2002, Rennie et al., 1999). Therefore, the results obtained could suggest that the types of lipids in the freeze-dried milk and infant formula possess different plastic and elastic properties that led to increased plastic deformation in the freeze-dried milk tablets during compression and consequently a greater extent of binding capability than the lipids in infant formula tablets.

#### 3.1.3 Friability test

An alternative method to determine the resistance of tablets to applied mechanical strength is friability. Unlike the breaking force test, the friability test determines the resistance of tablets to chipping and surface abrasion by rotating in a tumbler. It was found that only the freeze-dried milk-cinnarizine tablets in the presence and absence of PVPP had a friability of <1% (percentage weight loss of material after tumbling). Friability of this value indicates that the tablets are able to endure the rigours of handling in manufacture, transportation, dispensing and usage.

#### 3.1.4 Disintegration and dissolution tests

**Fig. 3:**
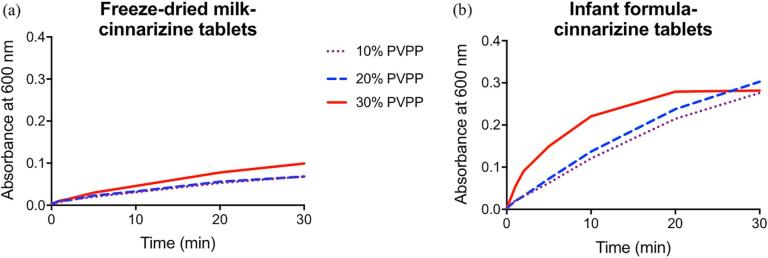
Turbidity as a function of time for (a) freeze-dried milk-cinnarizine tablet and (b) infant formula-cinnarizine tablet with increasing amounts of PVPP added.

Initially, three compression forces of 60, 250 and 440 kg were used to produce freeze-dried milk and infant formula tablets. Tablets produced with 250 and 440 kg compression forces displayed disintegration times greater than two hours and thus all subsequent batches of tablets were made with a compression force of 60 kg. As shown in Table 3, freeze-dried milk and infant formula tablets disintegrated completely after 41 and 33 min, respectively, in water. PVPP was added such that the mass of disintegrant was 30% of the mass of the freeze-dried milk/infant formula-drug mixture added to the press. Upon addition of the disintegrant, the disintegration time reduced significantly with the freeze-dried milk tablets displaying a disintegration time of 22 min and the infant formula tablets having a disintegration time of 6 min. Following the disintegration studies, dissolution experiments were performed to determine the release rate of cinnarizine from the tablets. Results in Fig. 3 show that freeze-dried milk and infant formula dispersed at a faster rate with increasing amount of disintegrant, PVPP. Moreover, complete dispersion of the tablets was observed when disintegrant was added in the case of the infant formula-cinnarizine tablets after 20–30 min. In contrast, although the disintegration rate did increase at a higher amount of disintegrant used for freeze-dried milk-cinnarizine tablets, incomplete dispersion of the tablets was seen by the absence of a plateau in turbidity after 30 min (Fig. 3a). The incomplete dispersion of the freeze-dried milk tablets could be due to the aggregation or denaturation of milk proteins during compression of the tablet or having different lipids to the infant formula, increasing the hydrophobic nature of the tablets.

As described in previous studies, the average volume-weighted mean diameter (D_4,3_) of homogenised milk is around 0.56–0.63 μm (Gallier et al., 2013, Clulow et al., 2018). In contrast, the average particle size was greater after dispersion of the freeze-dried milk tablets (average 5.86 μm) and infant formula tablets (average 2.0 μm). The larger particle size following the reconstitution of freeze-dried milk could be attributed to some aggregation of the fat droplets (Orubu et al., 2017). Moreover, the addition of PVPP to the system resulted in a larger average particle sizes, with D_4,3_ = 93.03 μm and 80.31 μm upon dispersion of the freeze-dried milk-cinnarizine tablets and infant formula-cinnarizine tablets, which is likely due to the swelling nature of PVPP.

### 3.2 Effect of tableting on self-assembly of milk/infant formula lipids

**Fig. 4:**
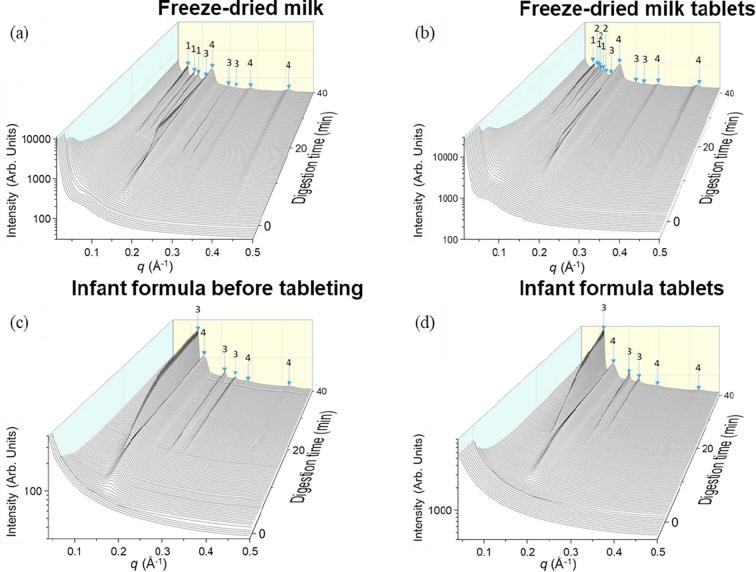
SAXS profiles for the digestion of (a) freeze-dried milk, (b) freeze-dried milk tablets, (c) infant formula before tableting and (d) infant formula tablets after the addition of lipase over 40 min at pH 6.5, 37 °C. “1″ represents the bicontinuous cubic *Im*3*m* phase, with peak *q* ratios of √2: √4: √6, “2” refers to the cubic *Pn*3*m* phase with peak *q* ratios of √2: √3: √4, “3” is annotated as hexagonal phase with peak *q* ratios of 1: √3: √4 and “4” represents the lamellar phase with peak *q* ratios of 1: 2: 3.

It has been previously reported that a series of liquid crystalline structures arising from self-assembly of milk lipids from bovine milk appear during digestion as a result of the self-assembly of TAG, DAG, MAG and FFA (Salentinig et al., 2013, Clulow et al., 2018). No significant differences were obtained in the types of liquid crystalline structures formed during digestion of commercial, raw, spray-dried, freeze-dried and frozen milk but their formation kinetics did change (Clulow et al., 2018). Specifically, the formation of the micellar cubic phase *Fd*3*m*, inverse hexagonal phase H_2_, and bicontinuous cubic phase *Im*3*m*, with coexisting lamellar soaps L_α_ were observed sequentially for these systems including freeze-dried milk (Clulow et al., 2018), the SAXS pattern of which is shown in Fig. 4a. However, differences in the liquid crystalline structures emerged following the tableting of freeze-dried milk. Since previous works have demonstrated that neither freeze-drying nor reconstitution of powdered milk led to appreciable changes in the structure of the digesting milk lipids, the process of tableting induced changes in the self-assembly of the milk lipids after redispersion and digestion. This resulted in the formation of a cubic *Pn*3*m* phase ~15 min into digestion. This is evident from scattering peaks at *q* = 0.061, 0.074 and 0.086 Å^−1^, indicated by the arrows marked 2 in Fig. 4b.

In contrast to the effect of tableting on milk, only minor changes in the self-assembled structures formed on digestion of the infant formula tablets were evident when compared to non-tableted infant formula, with both powdered and tableted forms displaying hexagonal and lamellar phases (Fig. 4c and d).

### 3.3 Effect of addition of cinnarizine and disintegrant on the self-assembly of milk/infant formula lipids

**Fig. 5:**
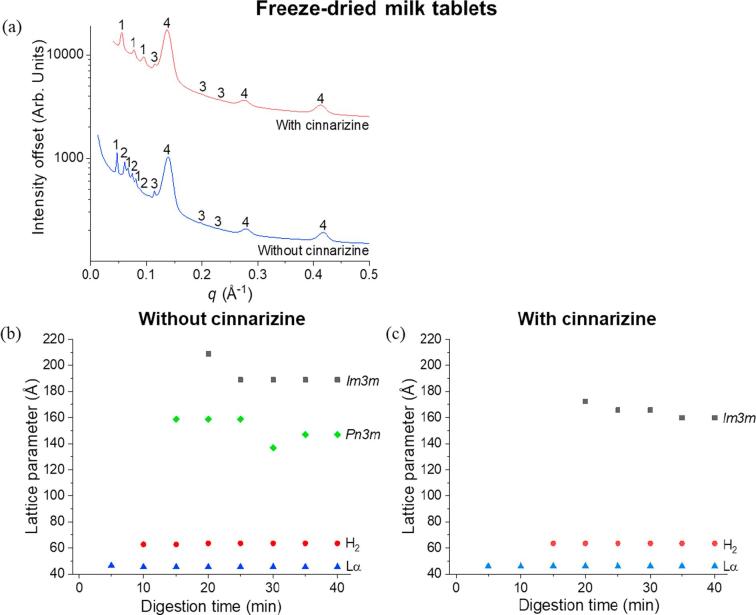
(a) SAXS profiles from the end of digestion of freeze-dried milk tablets (reproduced from Fig. 4b at time = 40 min) and freeze-dried milk-cinnarizine tablets. “1” represents the bicontinuous cubic *Im*3*m* phase, “2” refers to the cubic *Pn*3*m* phase, “3” is annotated as hexagonal and “4” represents the lamellar phase. Trends in lattice parameters as a function of digestion time of the phases formed from the self-assembly of milk lipids from (b) freeze-dried milk tablets and (c) freeze-dried milk-cinnarizine tablets.

The formulation of these dispersible tablets not only encompasses milk or infant formula but also includes the drug cinnarizine. It is important to determine whether there would be any modifications in the self-assembly of the milk lipids following the incorporation of cinnarizine. The SAXS profiles at the end of digestion of freeze-dried milk-cinnarizine tablets is shown in Fig. 5a. There was a reduction in the number of liquid crystalline phases from four phases without cinnarizine, to three phases with cinnarizine. In the latter, the additional cubic *Pn*3*m* phase was not observed and the liquid crystalline phases reverted back to those characteristically observed for unadulterated milk, with only lamellar, hexagonal and *Im*3*m* phases formed. As shown in Fig. 5b and c, the lattice parameters for the hexagonal and lamellar phases were similar irrespective of the presence of cinnarizine but the lattice parameters for the *Im*3*m* phase from the freeze-dried milk-cinnarizine tablets were found to be less than that from the freeze-dried milk tablets throughout the digestion process.

**Fig. 6:**
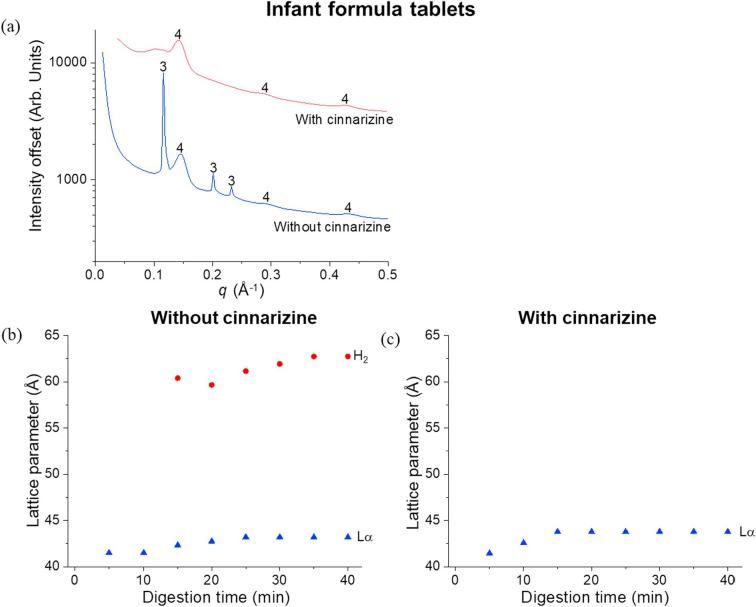
(a) SAXS profiles from the end of digestion of infant formula tablets (reproduced from Fig. 4d at time = 40 min) and infant formula-cinnarizine tablets. “3” is annotated as hexagonal and “4” represents the lamellar phase. Trends in lattice parameters as a function of digestion time of the phases formed from the self-assembly of lipids from (b) infant formula tablets and (c) infant formula-cinnarizine tablets.

Similar to freeze-dried milk tablets, addition of cinnarizine to the tablets in infant formula altered the liquid crystalline structure formation after 40 min digestion (Fig. 6a). It was not clear if the broad peak observed at a *q* value of around 0.107 Å^−1^ belonged to a hexagonal phase, as indexing could not be performed based on a single broad peak. Although the lattice parameters corresponding to the lamellar phase are similar with and without cinnarizine (Fig. 6b and c), the absence of the diffraction peak associated to the hexagonal phase again showed that addition of cinnarizine affects the self-assembly of the digesting lipids.

**Fig. 7:**
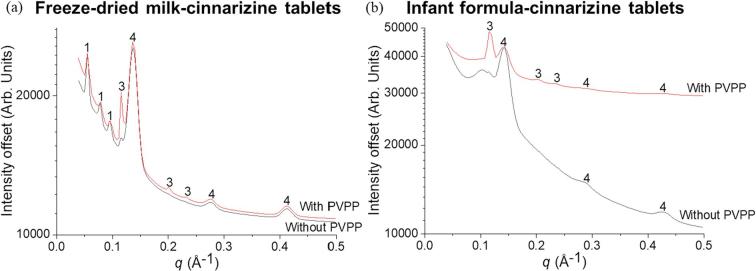
SAXS profiles from the end of digestion of (a) freeze-dried milk tablets with and without PVPP and (b) infant formula tablets with and without PVPP. “1” represents the bicontinuous cubic *Im*3*m* phase, “3” is annotated as hexagonal and “4” represents the lamellar phase.

Disintegration time is an important parameter that needs to be optimised in the development of a dispersible solid dosage form. As PVPP was added to the formulations to reduce the disintegration time from 41 min to 22 min for freeze-dried milk tablets and from 33 min to 6 min for infant formula tablets, it is important to understand whether addition of PVPP could affect the self-assembly of the milk/infant formula lipids. PVPP is generally recognised as an inert excipient, hence it was hypothesised that the liquid crystalline phases formed in the presence of PVPP would be the same as the earlier results i.e. without PVPP since the role of a disintegrant is to simply hasten the rate of dispersion. There were minor differences in the liquid crystalline phases formed with and without PVPP for the freeze-dried milk-cinnarizine tablets, where the presence of PVPP promoted the formation of the hexagonal phase in both types of milk (indicated as ‘3’ in both panels of Fig. 7). PVPP does have strong water sorption behaviour, so it is possible that it has a dehydrating effect of liquid crystalline systems which would explain the trend towards formation of the inverted hexagonal phase at the expense of the bicontinuous cubic phase.

### 3.4 Solubilisation of cinnarizine during digestion of dispersed freeze-dried milk and infant formula tablets

**Fig. 8:**
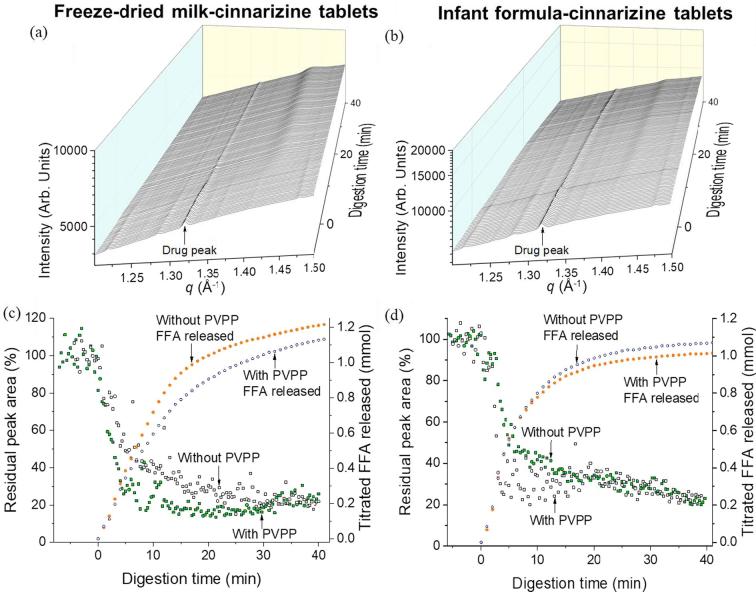
SAXS profiles during *in vitro* digestion of (a) freeze-dried milk-cinnarizine tablets and (b) infant formula-cinnarizine tablets between *q* = 1.20 and 1.50 Å^−1^. The peak at 1.32 Å^−1^ is characteristic for cinnarizine. Residual peak area of cinnarizine (represented by the green and black hollow squares) and titrated FFA released (indicated by the orange and blue hollow circles) of (c) freeze-dried milk-cinnarizine tablets with and without PVPP and (d) infant formula-cinnarizine tablets with and without PVPP. (For interpretation of the references to colour in this figure legend, the reader is referred to the web version of this article.)

The scattering profile of cinnarizine revealed a characteristic diffraction peak at *q* = 1.32 Å^−1^ present in both the freeze-dried milk and infant formula systems, consistent with previous reports (Vithani et al., 2018). It should be noted that drug was present in suspension at <2.5 mg/mL or 0.25 *w/v*% and was still detectable due to the sensitivity of the synchrotron SAXS technique to the presence of crystalline drug. Upon digestion (Fig. 8a and b), the intensity of the diffraction peak at *q* = 1.32 Å^−1^ decreased due to solubilisation of drug in the lipid digestion products. Integration of the diffraction peak intensity is shown in Fig. 8c and d, which indicates that solubilisation of crystalline drug was almost complete (∼80%) during the first 10–15 min of digestion for both the dispersed freeze-dried milk and infant formula tablets at equal fat content. The rate and extent of drug solubilisation was similar with and without PVPP (Fig. 8c and d).

## 4 Discussion

Paediatrics are categorised as a highly heterogeneous group due to their ages ranging from newborns to adolescents leading to differences in required dose, pharmacokinetics and bioavailability of drug. Dispersible tablets serve as a potential delivery platform for paediatrics due to convenience in dose control thereby enhancing patient compliance. This form of tablet is designed to disperse in practice in 10–20 mL of water and thus their disintegration time is critical in dictating their performance. While there are numerous strategies to decrease the disintegration and dissolution time such as implementing a low compression force and incorporating fast dissolving sugars and using effervescent excipients (Fu et al., 2004, Sandri et al., 2006), the use of a superdisintegrant was chosen in this study to enable dispersion in <20 min to allow digestion studies.

The initial disintegration time of the freeze-dried milk and infant formula tablets were approximately 41 and 33 min respectively. Addition of 30 *w/w*% PVPP resulted in a disintegration time of 22 and 6 min respectively (Table 2). Unlike other superdisintegrants where the mechanism of action varies depending on the excipient, PVPP incorporates the mechanism of wicking and swelling to enable rapid disintegration of the tablet (Pabari and Ramtoola, 2012). The porous structure of PVPP aids in wicking of water into the tablet, causing rapid volume expansion and hydrostatic pressures (Rowe, 2012). Together with the ability of PVPP to swell rapidly in water without gelling due to its high cross-link density, the wicking and swelling mechanisms cause interparticulate bonds in the tablet to rupture thereby promoting disintegration of the tablet.

Apart from the presence of different types of lipids in milk and infant formula, the difference in disintegration times could be a result of excipients that are prevalent in the infant formula used in this study but not in freeze-dried milk. Infant formula is designed to be redispersed from a solid powdered form by the inclusion of multiple excipients with strong surfactant properties, whereas milk is not designed to be redispersed but simply consumed in a liquid state. Although the disintegration times were still relatively long even with PVPP present, the primary aim of the study was to examine the influence of lipid digestion on the liquid crystalline phase formation in redispersed tablet mixtures and its effect on the solubility of entrained drugs. Other types of disintegrant could be explored to reduce the disintegration time with the aim of acquiring a disintegration time within 3 min in water at room temperature to produce a homogenous dispersion but this will be focus of future studies into tablet optimisation (WHO, 2011).

With different types of lipids present in freeze-dried milk and infant formula, it was anticipated that the self-assembly of lipids in milk and formula would differ (Christie and Clapperton, 1982). While triglycerides that dominate the lipid content before digestion are amorphous and do not self-assemble to form complex liquid crystalline structures in water, the more polar lipid digestion products (MAG and FFA) are generally amphiphilic and self-assemble in aqueous media. The crystalline structures formed can range from simple lamellar bilayers or more complex phases with internalised water channels such as hexagonal and bicontinuous cubic phases, which includes the observed *Im*3*m* and *Pn*3*m* phases (Hyde, 2001). It has been shown previously that subjecting milk to processes such as homogenisation, pasteurisation, freezing/melting and freeze-drying/reconstitution does not significantly affect the self-assembly of milk lipids, only their formation kinetics (Clulow et al., 2018). However, in this study it was found that compression to form a tablet induced subtle changes in the structure formation as observed by the appearance of an additional bicontinuous cubic phase, *Pn*3*m*, in freeze-dried milk tablets, after ~15 min of digestion. The reason behind the formation of this additional phase is unclear but the compression step may force interactions between the lipid and protein components not occurring in fresh milk, inducing slight changes in lipid structuring after redispersion (Fig. 4a and b). In comparison, the digestion of the dispersed infant formula tablets produced the same self-assembly phases as that of the non-tableted infant formula powder (Fig. 4c and d). The absence of an impact on self-assembly for the infant formula tablets as compared to the freeze-dried milk tablets further points to specific interactions between the non-lipid milk components and the lipids that are not present in the infant formula. In any case, the differences in phase behaviour between non-tableted and tableted milk were subtle, and indicate an overall robustness of the self-assembly process upon tableting of the two types of lipid formulations.

Cinnarizine is a poorly water-soluble weakly basic drug molecule (Khan et al., 2016). Physical characteristics of cinnarizine including hydrophobicity, surface charge and size of the drug molecules can influence the interaction with digested milk lipids such as the polar head groups, hydrophobic alkyl chains or both head groups and alkyl chains (Peetla et al., 2009, Mu et al., 2013, Mulet et al., 2013). The disappearance of the *Pn*3*m* phase in the digested freeze-dried milk-cinnarizine system and the hexagonal phase in the digested infant formula-cinnarizine system (Fig. 5, Fig. 6) would be an anticipated consequence of addition of a protonated amphiphilic drug that interacts with the lipid bilayers defining these structures. Nonetheless, liquid crystalline structures were still formed and could dictate transport of lipids and drug in these systems.

As discussed earlier, PVPP was chosen as a disintegrant for the tablet formulations. While there were no significant differences observed in the lipid liquid crystalline structures in the freeze-dried milk-cinnarizine and infant formula-cinnarizine tablets, the presence of the disintegrant promoted the formation of the hexagonal liquid crystalline phase in each case (Fig. 7). The growth of the hexagonal phase could be attributed to the hydrophobic side-groups of the polymeric disintegrant, which altered the thermodynamically favoured curvature of the lipid bilayers, causing the observed morphological changes (Bochicchio et al., 2017).

Previous studies have shown the effect of dispersing drug in milk and evaluating the characteristics of milk tablets. For instance, while Macheras et al., reported that drugs in freeze-dried milk dispersions display greater solubility than drugs in water alone (Macheras and Reppas, 1986, Macheras et al., 1990), Pinto et al., and Orubu et al., suggested that tablets containing different amounts of milk influence the performance of tablets, such as their tensile strengths and disintegration time (Pinto et al., 2016, Orubu et al., 2017). However, no previous studies on dispersible milk tablets have considered the effect of digestion of milk lipids on drug solubilisation. We have demonstrated in this study that drug solubilisation from freeze-dried milk/infant formula-drug tablets occurred following the digestion of milk lipids by implementing *in situ* monitoring of drug diffraction peak intensity in the small-angle X-ray scattering region (Fig. 8a and b). The decrease in intensity of the drug diffraction peaks during digestion could be due to a number of processes including dissolution, polymorphic transformations or conversion to an amorphous solid. The term ‘solubilisation’ is intended to reflect interaction with liberated fatty acids that removes the crystalline nature of the drug – the field is not yet settled on whether this process would represent true dissolution, or a conversion to an amorphous form that may or may not be solid. Absence of new peaks in the diffractograms attributable to drug alone precludes a polymorphic solid-solid transformation.

The conversion of drug from solid to solubilised form is somewhat at odds with the design of typical lipid-based formulations, where it is assumed that dissolution of drug from the crystalline form in the initial formulations is the limiting step. Many formulations are therefore designed to present a drug in an initially dissolved state and then maintain it in a dissolved state during dispersion and digestion. In the case of milk and infant formula, the drug begins in its solid crystalline form in the formulation and solubilisation is promoted by digestion of the entrained lipids, which is akin to the normal fatty food effect. In this case, the production of colloidal lipid structures during digestion acts as a reservoir for dissolution of drug and delivery to the intestinal sites of absorption. For poorly soluble weakly basic drugs, such as cinnarizine, the enhanced solubility in the digestion-derived fatty acids indicates this approach as a highly versatile dose form for these drugs.

In order to translate the milk tablets into a medicine, there are significant regulatory hurdles to overcome, in part due to the potential for variability in milk composition, which is not desirable from the point of view of ensuring consistent ‘medicine quality and performance’. However, demonstration of drug solubilisation during digestion of the infant formula tablet, a highly regulated infant food that undergoes stringent quality control and safety evaluation in paediatric populations provides a potential avenue to satisfy these requirements.

## 5 Conclusion

Dispersible milk-drug tablets were formulated using freeze-dried milk or infant formula and their tablet properties such as uniformity of weight, thickness, tensile strength, friability and disintegration time were evaluated. While minor differences in the tensile strength and friability of the freeze-dried milk and infant formula tablets were observed, the most crucial differences were in the disintegration times. Although none of the tablets disintegrated within 3 min as required of a dispersible tablet, the disintegration times were significantly reduced by the inclusion of the PVPP excipient suggesting that further improvements are possible. Tablets containing 30 *w/w*% PVPP disintegrant where the tablets could be dispersed within 20 min, formed particles with average volume-weighted diameters of 93.03 μm (freeze-dried milk-cinnarizine tablets) and 80.31 μm (infant formula-cinnarizine tablets) on dispersion. *In situ* monitoring of the lipid liquid crystalline structures formed during the digestion of the dispersed tablets revealed that tableting induced only minor changes in structure formation, where an additional cubic phase was evident on tableting of the freeze-dried milk that was not evident on digestion of the non-tableted milk formulation. This is likely due to compression of the freeze-dried milk matrix but further studies are required to elucidate the processes occurring with the self-assembly of lipids. The lipophilic drug, cinnarizine, was solubilised as a result of the formation of the digestion products from the milk fat. This highlights that dispersible freeze-dried milk/infant formula-drug tablets can be formulated and once redispersed, the lipids digest similar to off-the-shelf milk. Future studies will focus on improving the dispersibility of such tablets and measuring the influence of altering the milk fat content and type on the performance of milk-drug tablets for oral drug delivery.

## References

[b0005] Bochicchio D., Panizon E., Monticelli L., Rossi G. (2017). Interaction of hydrophobic polymers with model lipid bilayers. Sci. Rep..

[b0010] Christie W.W., Clapperton J.L. (1982). Structures of the triglycerides of cows' milk, fortified milks (including. infant formulae), and human milk. Int. J. Dairy Technol..

[b0015] Clulow A.J., Salim M., Hawley A., Boyd B.J. (2018). A closer look at the behaviour of milk lipids during digestion. Chem. Phys. Lipids.

[b0020] Davies E.H., Tuleu C. (2008). Medicines for Children: a Matter of Taste. J. Pediatr..

[b0025] Feeney O.M., Crum M.F., McEvoy C.L., Trevaskis N.L., Williams H.D., Pouton C.W. (2016). 50 years of oral lipid-based formulations: provenance, progress and future perspectives. Adv. Drug Deliv. Rev..

[b0030] Fu Y., Yang S., Jeong S.H., Kimura S., Park K. (2004). Orally fast disintegrating tablets: developments, technologies, taste-masking and clinical studies. Crit. Rev. Ther. Drug Carrier Syst..

[b0035] Gallier S., Cui J., Olson T.D., Rutherfurd S.M., Ye A., Moughan P.J. (2013). In vivo digestion of bovine milk fat globules: effect of processing and interfacial structural changes. I. Gastric digestion. Food Chem..

[b0040] Hyde S.T. (2001). Identification of lyotropic liquid crystalline mesophases. Handbook of Applied Surface and Colloid Chemistry.

[b0045] Jensen R.G. (1995). Handbook of Milk Composition.

[b0050] Kamal S.S., Kaur D., Singh S., Sharma A., Katual M.K., Garg A.K. (2016). An investigative and explanatory review on use of milk as a broad-spectrum drug carrier for improvement of bioavailability and patient compliance. J. Young Pharm..

[b0055] Kaukonen A.M., Boyd B.J., Charman W.N., Porter C.J. (2004). Drug solubilization behavior during *in vitro* digestion of suspension formulations of poorly water-soluble drugs in triglyceride lipids. Pharm. Res..

[b0060] Khan J., Rades T., Boyd B.J. (2016). Lipid-based formulations can enable the model poorly water-soluble weakly basic drug cinnarizine to precipitate in an amorphous-salt form during *in vitro* digestion. Mol. Pharmaceutics.

[b0065] Kirby N.M., Mudie S.T., Hawley A.M., Cookson D.J., Mertens H.D.T., Cowieson N. (2013). A low background-intensity focusing small-angle X-ray scattering undulator beamline. J. Appl. Crystallogr..

[b0070] Krause J., Breitkreutz J. (2008). Improving drug delivery in paediatric medicine. Pharm. Med..

[b0075] Kushner J., Langdon B.A., Hicks I., Song D., Li F., Kathiria L. (2014). A quality-by-design study for an immediate-release tablet platform: examining the relative impact of active pharmaceutical ingredient properties, processing methods, and excipient variability on drug product quality attributes. J. Pharm. Sci..

[b0080] Liu Z., Wang J., Cocks B.G., Rochfort S. (2017). Seasonal variation of triacylglycerol profile of bovine milk. Metabolites.

[b0085] Macheras P.E., Koupparis M.A., Antimisiaris S.G. (1990). Drug binding and solubility in milk. Pharm. Res..

[b0090] Macheras P.E., Reppas C.I. (1986). Studies on freeze-dried drug-milk formulations II: effect of regenerated fluid volume on nitrofurantoin bioavailability. J. Pharm. Sci..

[b0095] Michalski M.C., Briard V., Michel F. (2001). Optical parameters of milk fat globules for laser light scattering measurements. Le Lait, INRA Ed..

[b0100] Mu H., Holm R., Müllertz A. (2013). Lipid-based formulations for oral administration of poorly water-soluble drugs. Int. J. Pharm..

[b0105] Mulet X., Boyd B.J., Drummond C.J. (2013). Advances in drug delivery and medical imaging using colloidal lyotropic liquid crystalline dispersions. J. Colloid Interface Sci..

[b0110] Nunn T., Williams J. (2005). Formulation of medicines for children. Br. J. Clin. Pharmacol..

[b0115] Orubu S.E., Hobson N.J., Basit A.W., Tuleu C. (2017). The Milky Way: paediatric milk-based dispersible tablets prepared by direct compression – a proof-of-concept study. J. Pharm. Pharmacol..

[b0120] Özkan N., Walisinghe N., Chen X.D. (2002). Characterization of stickiness and cake formation in whole and skim milk powders. J. Food Eng..

[b0125] Pabari R.M., Ramtoola Z. (2012). Effect of a disintegration mechanism on wetting, water absorption, and disintegration time of orodispersible tablets. J. Young Pharm..

[b0130] Peetla C., Stine A., Labhasetwar V. (2009). Biophysical interactions with model lipid membranes: applications in drug discovery and drug delivery. Mol. Pharmaceutics.

[b0135] Pinto J.T., Brachkova M.I., Fernandes A.I., Pinto J.F. (2016). Evaluation of the ability of powdered milk to produce minitablets containing paracetamol for the paediatric population. Chem. Eng. Res. Des..

[b0140] Pouton C.W. (2006). Formulation of poorly water-soluble drugs for oral administration: physicochemical and physiological issues and the lipid formulation classification system. Eur. J. Pharm. Sci..

[b0145] Rennie P.R., Chen X.D., Hargreaves C., Mackereth A.R. (1999). A study of the cohesion of dairy powders. J. Food Eng..

[b0150] Rowe R. (2012). Handbook of Pharmaceutical Excipients.

[b0155] Salentinig S., Phan S., Khan J., Hawley A., Boyd B.J. (2013). Formation of highly organized nanostructures during the digestion of milk. ACS Nano.

[b0160] Salim M., Khan J., Ramirez G., Clulow A.J., Hawley A., Ramachandruni H. (2018). Interactions of artefenomel (OZ439) with milk during digestion: insights into digestion-driven solubilization and polymorphic transformations. Mol. Pharmaceutics.

[b0165] Sandri G., Bonferoni M.C., Ferrari F., Rossi S., Caramella C. (2006). Differentiating factors between oral fast dissolving technologies. Am. J. Drug Deliv..

[b0170] Soyeurt H., Dardenne P., Dehareng F., Lognay G., Veselko D., Marlier M. (2006). Estimating fatty acid content in cow milk using mid-infrared spectrometry. J. Dairy Sci..

[b0175] Topaloğlu Y., Yener G., Gönüllü U. (1999). Inclusion of ketoprofen with skimmed milk by freeze-drying. Il Farmaco..

[b0180] USP. 2002. <905> Uniformity of dosage units. United States Pharmacopeia.

[b0185] USP. 2011. <711> Dissolution. United States Pharmacopeia.

[b0190] USP. 2012. <1217> Tablet breaking force. United States Pharmacopeia.

[b0195] USP. 2016. <701> Disintegration. United States Pharmacopeia.

[b0200] USP. 2016. <1216> Tablet friability. United States Pharmacopeia.

[b0205] Vithani K., Hawley A., Jannin V., Pouton C., Boyd B.J. (2018). Solubilisation behaviour of poorly water-soluble drugs during digestion of solid SMEDDS. Eur. J. Pharm. Biopharm..

[b0210] Warren D.B., Anby M.U., Hawley A., Boyd B.J. (2011). Real time evolution of liquid crystalline nanostructure during the digestion of formulation lipids using synchrotron small-angle X-ray scattering. Langmuir.

[b0215] WHO (2010). Development of Paediatric Medicines: Points to Consider in Pharmaceutical Development.

[b0220] WHO (2011). Revision of monograph on tablets.

[b0225] Williams H.D., Sassene P., Kleberg K., Bakala-N'Goma J.C., Calderone M., Jannin V. (2012). Toward the establishment of standardized *in vitro* tests for lipid-based formulations, part 1: method parameterization and comparison of *in vitro* digestion profiles across a range of representative formulations. J. Pharm. Sci..

