# Supplementary material for: Revisiting dispersible milk-drug tablets as a solid lipid formulation in the context of digestion
Source: Int J Pharm. 2019 Jan 10;554:179–89. doi: 10.1016/j.ijpharm.2018.10.069 (PMC6328708; doi:10.1016/j.ijpharm.2018.10.069)
Supplement: Supplementary Data 1 [file mmc1.docx]

# Supporting information

**Revisiting dispersible milk-drug tablets as a solid lipid formulation in the context of digestion**

**Syaza Y. Binte Abu Bakar^1^, Malinda Salim^1^, Andrew J. Clulow^1^, Adrian Hawley^2^ and Ben J. Boyd^3*^**

*^1^Drug Delivery, Disposition and Dynamics, Monash Institute of Pharmaceutical Sciences, Monash University, 381 Royal Parade, Parkville, Victoria 3052, Australia*

*^2^SAXS/WAXS beamline, Australia Synchrotron, ANSTO, 800 Blackburn Road, Clayton, Victoria 3169, Australia*

*^3^ARC Centre of Excellence in Convergent Bio-Nano Science and Technology, Monash Institute of Pharmaceutical Sciences, Monash University, 381 Royal Parade, Parkville, Victoria 3052, Australia.*

__________________________________________________________________________________________

Supplementary material, especially figures as referred in the manuscript is detailed below:

**Test for lipase activity**

The enzymatic activity of the lipase was determined using the tributyrin (TBU) test. 6 g of tributyrin (5.8 mL) was dispersed in 50 mM digestion buffer (18 mL) and allowed to stir for 15 minutes prior to the addition of 2 mL of lipase (434 mg lipase in 2 mL buffer). The pH of the buffer was adjusted to 7.5 using 1 M or 2 M NaOH at 37 °C and left to run for 10 min.

**Table S1.** Physicochemical properties of cinnarizine (Information adapted from Tokumura et al., 1987, Branchu et al., 2007 and Parikh et al., 2006).

| **Chemical structure** | 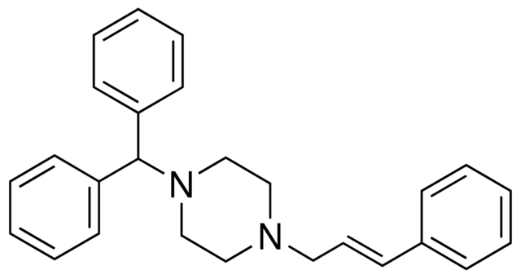 |
| --- | --- |
| **Log P** | 5.8 |
| **Weakly basic** | pK_a1_ = 2.0, pK_a2_ = 7.5 |
| **Solubility** | 0.29 mg/mL in 0.1 M HCl  0.002 mg/mL in phosphate buffer pH 7.2 |

**Figure S1**. Comparison of milk-drug tablet and AUS$2 coin in terms of (a) diameter and (b) thickness.


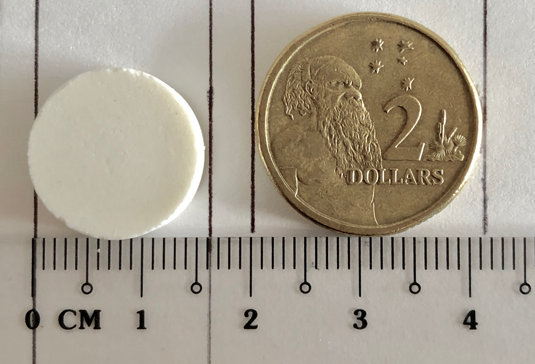

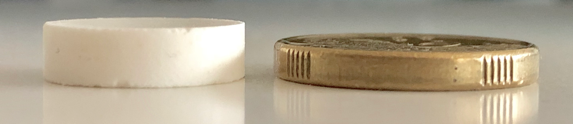


(a)

(b)


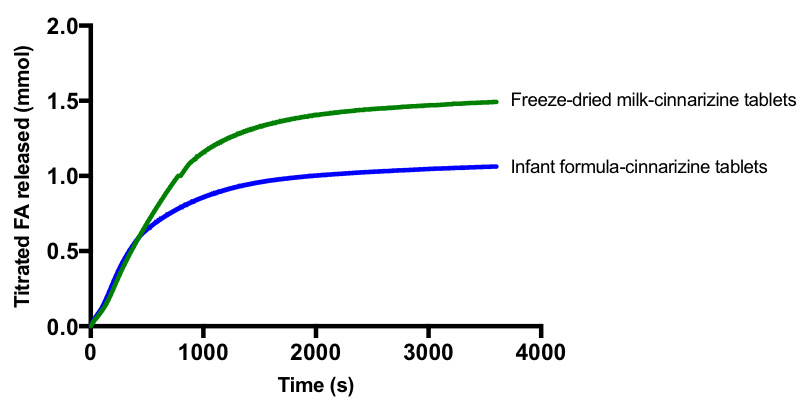


**Figure S2**. Titrated fatty acids released during an hour digestion for freeze-dried milk-cinnarizine and infant formula-cinnarizine tablets.

**Figure S3.** The X-ray diffratogram of cinnarizine powder was recorded at the synchrotron and the most prominent peak was selected where q = 1.32 Å^-1^.
